# Chikungunya: From Hypothesis to Evidence of Increased Severe Disease and Fatalities

**DOI:** 10.3390/v17010062

**Published:** 2025-01-03

**Authors:** Carlos Brito, Melissa Barreto Falcão, Maria de Fatima Pessoa Militão de Albuquerque, Thiago Cerqueira-Silva, Maria Glória Teixeira, Rafael Freitas de Oliveira Franca

**Affiliations:** 1 Programa de Pós-Graduação em Medicina Tropical, Centro de Ciências Médicas, Universidade Federal de Pernambuco (UFPE), Recife 50740-465, Brazil; cbritoc@gmail.com; 2 Departamento de Imunologia, Instituto Autoimune de Pesquisa, Recife 52011-040, Brazil; 3 Universidade Estadual de Feira de Santana (UEFS), Feira de Santana 44036-900, Brazil; melissa.falcao@hotmail.com; 4 Instituto Aggeu Magalhães, Fundação Oswaldo Cruz, Recife 50740-465, Brazil; militaofatima@gmail.com; 5 Laboratório de Medicina e Saúde Pública de Precisão, Fundação Oswaldo Cruz, Salvador 40296-710, Brazil; thiago.c.silva@fiocruz.br; 6 Instituto de Saúde Coletiva, Universidade Federal da Bahia (UFBA), Salvador 40026-010, Brazil; t.gloria@hotmail.com; 7 Fundação Oswaldo Cruz—Fiocruz São Paulo, Ribeirão Preto 14049-900, Brazil

**Keywords:** chikungunya, severe disease, mortality, epidemiology

## Abstract

Chikungunya virus infection often manifests as an acute, self-limiting febrile illness, with arthralgia and musculoskeletal symptoms being the most commonly reported. Arthralgia can persist for months or even years, and approximately 50% of cases progress to chronic conditions. However, recent outbreaks have revealed a rising number of severe cases and fatalities. This review examines evidence from the past decade that suggests a higher incidence of severe chikungunya virus (CHIKV) infections and increased mortality rates, challenging official reports and guidelines from many countries. The literature review includes case reports, series, and studies with comparison groups to assess whether CHIKV-related mortality is underreported. Evaluating excess mortality involves analyzing consistent findings across different regions, biological plausibility, and systemic manifestations that contribute to severe outcomes. These findings aim to expand disease classifications in international guidelines and raise awareness among healthcare professionals to better identify severe CHIKV cases and related deaths. Studies were identified through PubMed using the search terms “chikungunya”, “death”, “severe”, “pathogenesis”, and “pathophysiology”.

## 1. Introduction

Chikungunya virus (CHIKV), an arbovirus from the *Togaviridae* family, Alphavirus genus, was first reported in 1952 in Tanzania [1,2]. However, it stayed restricted to the African continent until 2005, when significant outbreaks were documented across the Indian Ocean islands, extending to the majority of Indian states in the subsequent years. In December 2013, the virus reached the Americas, causing an outbreak in the Caribbean with a prevalence of the CHIKV Asian genotype, spreading to 38 other locations in the Americas. The total number of cases in the region, until the first quarter of 2024 was 4,105,503, with Brazil accounting for almost half of them 1,929,121 (47%). Over a period of 10 years, ten million cases were reported worldwide [3].

CHIKV infection typically presents as an acute febrile illness, with arthralgia and musculoskeletal manifestations as the most commonly described symptoms. Arthralgia can persist for months to years, with approximately 50% of cases leading to chronicity. Extra-articular manifestations and severe cases have been described as atypical and are considered rare clinical events [4]. CHIKV demonstrates notably high attack rates, with a considerably greater proportion of symptomatic cases compared to other arboviruses. Attack rates can reach 35 to 75% of a population in a single outbreak [3,5]. In the Réunion Island outbreak, a surveillance study confirmed 16,050 cases through laboratory testing and estimated 244,000 cases, corresponding to a 35% attack rate [6,7]. Approximately 75 to 95% of individuals infected with CHIKV may experience a symptomatic illness [3,4].

In this review, we present an update on the evidence from the past decade that supports a higher frequency of severe forms of the disease and an increased risk of death. This evidence contrasts with the official notification report from many countries, the concepts described in guidelines from official institutions, and the perception within part of the medical community that these cases are rare or infrequent. To demonstrate that there is more mortality than has been notified, we reviewed the literature, from case reports and case series to studies with comparison groups. Furthermore, demonstrating whether there is an excess mortality associated with CHIKV requires a careful analysis to confirm this hypothesis. This includes examining the consistency of results across different locations, the biological plausibility from experimental and pathophysiological studies, the identification of systemic clinical manifestations that may determine or contribute to severe forms and fatal outcomes, and the comparison of risks between infected and healthy individuals, among other findings published in the literature. These findings will facilitate the incorporation of new classifications of the disease spectrum into international guidelines, extending beyond joint manifestations. This, in turn, will raise awareness among healthcare professionals for the detection of severe cases and related deaths. For this review, studies were identified using PubMed with the following search terms: “chikungunya”, “death”, “severe”, “pathogenesis”, and “pathophysiology”, with and between them.

## 2. The Hypothesis of a Disease Extending Beyond Joint Involvement

Over the past decade, accumulating scientific evidence has reinforced that an excess mortality may be associated with CHIKV infection. In 2014, CHIKV was introduced in Brazil, which became the country with the highest number of cases in South America, with a total of 773,000 cases in 2014 and successive outbreaks in the following years. The first cases of CHIKV in Brazil occurred in the second half of 2014 in the cities of Oiapoque, Amapá (North Brazil), and almost simultaneously in Feira de Santana, Bahia (Northeast) [8,9]. In the following year, CHIKV reached a total of 265,554 cases, with 230,582 occurring in the Northeast region. Pernambuco state accounted for 20% of the cases in the Northeast, with an incidence rate of 510.6 cases per 100,000 inhabitants [10].

The first evidence of underreporting of possible CHIKV-associated mortality emerged in March 2016. In the Northeast of Brazil, healthcare professionals reported an increase in the number of severe cases and fatalities among patients with CHIKV symptoms, despite the small number of fatal outcomes recorded in official statistics. These findings alerted the authorities to the potential for underreporting and suggested that severe cases may be more common than previously estimated [11,12]. Indeed, Brito et al. in 2017 proposed that CHIKV-related deaths went largely unrecognized within the healthcare system due to the limited understanding of the symptoms and complications associated with CHIKV infection. This is unsurprising, considering that CHIKV had only recently been introduced in the Americas. The authors suggested that secondary causes of death, such as cardiac, respiratory, and metabolic syndromes, were being reported without any reference to CHIKV infection as the primary or triggering cause [12]. Given that CHIKV was not listed as the primary cause of death, the authors conducted a comprehensive survey of all deaths that occurred in 2016 and compared them with numbers from the previous years. They identified an excess of deaths considering the average of the four previous years, with the peak in the number of deaths coinciding with the peak of the CHIKV cases in Northeastern Brazil [10]. After this, other states were evaluated, and the findings were consistent with excess mortality during the chikungunya epidemic [13]. In fact, severe CHIKV disease has already been described in the literature [7,14]. Renault et al. in 2007 reported 203 deaths due to CHIKV infection on Réunion Island, along with cases of hospitalization related to respiratory failure, decompensated heart failure, meningoencephalitis, severe hepatitis, and acute kidney failure, among others [7]. Economopoulou et al. in 2009 reported 610 adults with atypical presentations of CHIKV, including cardiovascular manifestations (heart failure, arrhythmia, myocarditis, acute coronary disease), neurological disorders (encephalitis, meningoencephalitis, seizures, Guillain–Barré syndrome), prerenal insufficiency, developing pneumonitis, and respiratory failure [14]. Lastly, Cerqueira-Silva et al. reported an increased risk of death within 84 days after symptom onset of chikungunya, with an additional 146 deaths per 100,000 cases; among the cause of death were diabetes and ischemic heart and kidney diseases [15].

Despite this, international guidelines and review articles published between 2008 and 2024 considered this event as atypical and unusual. The Pan American Health Organization (PAHO) in its manual Guidelines for the Clinical Diagnosis and Treatment of Dengue, Chikungunya, and Zika (available at https://www.paho.org/en/documents/guidelines-clinical-diagnosis-and-treatment-dengue-chikungunya-and-zika (accessed on 1 September 2023)) considers that serious forms of the disease with atypical manifestations may rarely occur. Consequently, other guidelines began to replicate this perception, portraying chikungunya as a less severe disease with a lower risk of fatal outcomes. In its 2015 bulletin, the PAHO considered that the case fatality rate in Latin America was lower than reported in the literature; however, these data have since been questioned. As of 2023, the organization’s website states that CHIKV-related deaths are very rare and almost always associated with other existing health conditions (available at https://www3.paho.org/data/index.php/en/mnu-topics/chikv-en/550-chikv-weekly-en.html (accessed on 1 September 2023). Thus, CHIKV case classification and management guidelines have primarily been based on the intensity and duration of joint symptoms, with little to no emphasis on severe clinical forms associated with systemic disease and organ involvement [16,17].

Review articles have also increasingly reinforced and replicated the perception that CHIKV poses a lower risk of fatal outcomes [3,18,19,20]. In a comprehensive review of the disease’s epidemiology in the Americas, Yactayo highlighted that CHIKV infection is typically non-fatal [18]. Similarly, Khongwichit et al., when analyzing epidemics in Asia, observed that CHIKV infection rarely progresses to severe or life-threatening forms. Deaths are infrequent and are usually associated with pre-existing health conditions or severe clinical manifestations in the elderly, children, or immunocompromised patients [19].

## 3. Excess Mortality—Temporarily Associated and Consistent Reported Among Different CHIKV Outbreaks

During the outbreak of 2016 in Brazil, suspecting that there were more fatal cases than those reported by the official system, Brito et al. in 2016 evaluated public hospital deaths from all causes in the official notification system for that year and compared them with the numbers (absolute and percentage differences) of the previous four years. There was an excess of 4235 deaths in 2016 compared to the average of the previous four years (20% increase), with the largest differences occurring from January to April, which coincided with the CHIKV outbreak peak. In Pernambuco state, Northeastern Brazil, during the first four months of 2016, there were a total of 2919 excess deaths (compared to the average of previous years) [10,12]. Additionally, in another study in Brazil, Freitas et al. calculated the expected mortality rates based on the monthly average of the age-standardized mortality rate for the same months between 2011 and 2013 (pre chikungunya introduction) and calculated the changes in mortality rate per 100,000 inhabitants and the percentage variation (%) in each region. The excess mortality rates per 100,000 inhabitants and the excess deaths in the four critical regions studied were Pernambuco (47.9 and excess deaths = 4505), corresponding to a 21% increase in deaths; Rio Grande do Norte (42.5 and 1478); Southern Bahia (32.0 and 546); and Northern Bahia (30.0 and 339) [13].

Independent studies performed in other locations of America outside Brazil also observed similar results. In Jamaica, the analysis of the CHIKV epidemic in 2014 identified 2499 excess deaths, leading to an overall increase in mortality of 91.9 per 100,000 inhabitants. Mortality was higher among the elderly but was also identified, in a minor proportion in the younger groups, even in children under five years. The excess mortality rate in the 50 to 59 age group was 104.6 per 100,000 inhabitants, and among those over 70, it was 998.6 per 100,000 inhabitants (~1%), with excess mortality coinciding with the peak of CHIKV cases reported during the outbreak. In Guadeloupe and Martinique, a total of 153,400 cases of CHIKV were reported in 2014, with an estimated 306,800 symptomatic cases and 160 deaths. When evaluating the excess of mortality, compared to three years preceding the outbreak, the authors found higher numbers of probable deaths than officially reported. Pearson’s correlation analysis showed a strong correlation between monthly excess deaths and reported CHIKV cases (R = 0.81, *p* < 0.005) and with a one-month interval (R = 0.87, *p* < 0.001). There was also a strong correlation between monthly hospitalization rates for CHIKV and excess deaths with a one-month interval (R = 0.87, *p* < 0.0005). These delays may be related to deaths occurring in the subacute phases of the disease. The epidemic peaked during the month of highest mortality, returning to normal levels shortly after the end of the CHIKV wave [21].

During the 2014 epidemic in the Dominican Republic, six deaths from chikungunya were officially recorded. However, when evaluating total deaths reported in the same period, an excess of 2853 deaths was observed compared to the two years before the outbreak. There was a strong correlation between the monthly excess deaths and CHIKV cases (Pearson’s r = 0.89), with the highest excess deaths among the elderly, but also with excess deaths in children under five and adults over 40 [22]. In Puerto Rico during the 2014–2015 epidemic where 28,327 cases and 31 deaths were officially reported, 1310 excess deaths were identified during the peak months of the CHIKV wave compared to previous years (no other major outbreaks were reported in the same period). The age pattern of affected individuals remained consistent, with the elderly being most frequently affected. This finding was reinforced in the matched cohort and self-controlled case series conducted in Brazil, demonstrating evidence of increased risk of death in those aged more than 60 years compared to the younger group [23]. However, a significant proportion of cases also occurred among those aged 24 to 55, indicating that deaths potentially associated with chikungunya are not exclusive to the elderly. Similar to the results published for the Mauritius islands, there was a one-month interval between the peak of CHIKV cases and the peak of excess deaths and also identified an excess of deaths due to cardiovascular causes and diabetes. These findings support the hypothesis that deaths related to CHIKV infection are due to both direct causes and the decompensation of comorbidities. Despite the attention drawn from the high volume of publications during the Brazilian epidemic showing excess deaths, previous publications have already been published from other outbreaks. In 2008, Mavalankar D. reported a total of 2944 excess deaths in Ahmedabad in India in 2006. The authors attributed this discrepancy to failures in the official surveillance system to classify deaths [24]. On the Mauritius Islands in India, Beesoon et al. in 2008 conducted a similar analysis and reported that death rates on Mauritius Island during the chikungunya epidemics in March, April, and May of 2006 were significantly higher than expected (*p* < 0.01 for all three months) compared to the ten years before the epidemic, corresponding to 743 excess deaths during these months [25]. Overall, these studies consistently identify an excess in mortality, reinforcing the temporality of outcomes with the months when chikungunya cases occurred. They also demonstrate a consistency of results across different regions affected by the epidemics. This contrasts with official data, which recorded a small number of deaths associated with infection. These results supported the hypothesis that surveillance could be flawed and that the lack of records might be related to unawareness and the failure of medical professionals to identify fatal outcomes during the epidemic. Table 1 summarizes the documented excess mortality across various CHIKV outbreaks.

## 4. Biological Plausibility—Systemic Clinical Manifestations That Determine or Contribute to Severe Forms and Fatal Outcomes

Reports of systemic manifestations, severe forms, and fatalities are still considered unusual by many publications, being classified as atypical CHIKV manifestations until recently [18,19,20,28]. Descriptions of systemic involvement leading to CHIKV severe disease and death have been reported over the past two decades from case reports, case series, and hospital-based studies [29,30,31,32,33]. Renault et al. reported that during the 2005–2006 CHIKV epidemic on Réunion Island, most of the 123 severe cases requiring hospitalization were related to respiratory failure; decompensated heart failure; or neurological, hepatic, or kidney impairment. Of the 203 fatal cases reported during the epidemic, 121 (60%) were identified as having CHIKV infection as the primary cause of death, while the remaining cases were listed as possibly associated with CHIKV [7].

In another study from the 2005–2006 outbreak on Réunion Island, among 610 patients hospitalized with confirmed CHIKV infection, the major causes for hospitalization were cardiovascular disease (37%), neurological manifestations (24%), kidney failure (20%), pneumonia (17%), and respiratory failure (8%). Although 89% of these patients had a history of pre-existing medical conditions, some complications were not related to their previous comorbidities. Among the 120 patients hospitalized for renal failure, 66% did not have a prior history of kidney disease. Of the 44 cases of arrhythmia, 63% had no cardiovascular history, and of the 131 cases with glycemic level changes, 20% were diagnosed with diabetes mellitus for the first time [14]. In the epidemics affecting the Americas from 2013 onwards, reports of severe complications among hospitalized patients also emerged. Crosby et al., during the 2013–2014 epidemic in Guadeloupe and Martinique, reported 65 cases of patients admitted to intensive care units, with the most frequent complications being neurological conditions such as encephalitis, Guillain–Barré syndrome, and septic shock. In patients with severe sepsis or septic shock, blood cultures were negative, suggesting that the manifestations were caused by CHIKV infection [33].

Shock as an event leading to fatal outcomes has also been reported by other authors [34,35]. In a study involving 42 patients hospitalized with severe CHIKV, 25 patients presented with severe sepsis. The mortality rate among patients who developed CHIKV sepsis was significantly higher than among those who experienced other severe manifestations (48% vs. 3%, *p* < 0.001). The cause of death in these patients was septic shock secondary to CHIKV systemic infection, with no other associated infections identified in either blood or urine cultures [35]. In Ceará, Northeast Brazil, Simião et al. in 2019 analyzed chikungunya deaths between 2016 and 2018, with approximately 200,000 CHIKV reported cases and 245 deaths officially confirmed, including 79 cases subjected to autopsy. The authors reported that respiratory failure (36.7%), septic shock (8.9%), and hypovolemic shock (7.6%) were the leading immediate causes of death [36]. de Souza et al. investigated deaths using OMICs (metabolomic and proteomic) and found that CHIKV was able to infect multiple organs, even impairing the integrity of the blood–brain barrier and causing damage to the central nervous system [37]. Altogether, these reports have accumulated new evidence reinforcing that organ damage is more relevant and frequent than previously described.

## 5. Chikungunya Virus Associated Neurological Symptoms

Neurological manifestations associated with CHIKV infection were initially described as uncommon or atypical, with the first reports of CHIKV-associated neurological cases occurring in India in 1964 [38]. However, neurological manifestations have become progressively more frequently reported as novel outbreaks have been reported. Since 2005, in the Indian Ocean islands, the Caribbean region, and South America, there has been a significant increase observed in the numbers of neurological cases associated with CHIKV infection [7,33,38,39,40]. In a systematic review, Mehta et al. identified 856 cases of neurological manifestations associated with chikungunya infection. Of these, five publications were from 1964 to 1971, and the remaining 89 articles were published from 2005 onwards. The authors comprehensively described 856 cases of neurological manifestations associated with CHIKV infection. The most frequent neurological symptoms included encephalopathy, encephalitis, myelopathy, myelitis, encephalomyelopathy, myeloneuropathy, Guillain–Barré syndrome, acute disseminated encephalomyelitis, and neuro-ocular diseases (uveitis, retinitis, optic neuritis) [41,42,43]. Gérardin et al. reported, in a retrospective cohort study, 57 cases with neurological manifestations associated with CHIKV between 2005 and 2006 in the Réunion Islands, including 27 cases of encephalitis, representing a cumulative incidence rate of 8.6 cases per 100,000 inhabitants and a case fatality rate of 16.6% [44]. Brito et al. conducted a prospective cohort study at a neurology hospital in Northeast Brazil during the ZIKV and CHIKV epidemics of 2015 and 2016. They reported 148 confirmed cases of neurological diseases associated with arboviral infections. Among these, CHIKV infection was the most frequent cause, present in 55 (56%) of 98 mono-infections and in all 50 co-infection cases. In this study, CHIKV infection was more frequently associated with central nervous system (CNS) disease (26 [47%] of 55 patients with CHIKV infection versus six [15%] of 41 with Zika infection cases; *p* = 0.0008). Myelitis (12 [22%] patients) accounted for the most commonly reported symptom. However, CHIKV neurological manifestations had a broad spectrum, with nine (16%) presenting peripheral nervous system (PNS) disease (including seven [13%] with Guillain–Barré syndrome), three (5%) with mixed CNS and PNS disease, nine (16%) with cranial neuropathies (including two [4%] with abducens nerve paresis), and eight (15%) with other neurological disorders [43]. Another study conducted in Southeast Brazil identified 122 cases of neurological diseases associated with arbovirus infections. CHIKV was the most frequently identified cause, present in 57.1% of confirmed cases [45]. Due to the high frequency of neurological disease associated with CHIKV, the authors suggest that CHIKV should be investigated in all patients presenting with acute neurological disease in endemic areas [41,42]. In fact, in Brazil, the Ministry of Health implemented specific guidelines at the national level for the surveillance of neuroinvasive diseases associated with arbovirus infection. Therefore, despite the relevance and frequency of neurological manifestations, diagnosing neuroinvasive arboviral infections remains a public health challenge, particularly in low- and middle-income countries with limited resources, leading to underreporting.

## 6. Cardiovascular Manifestations

Cardiovascular involvement has been described as one of the most frequent manifestations among severe cases and with fatal outcomes. During the 2013–2015 CHIKV outbreak in Martinique and Guadeloupe, out of 1836 hospitalized cases, 361 were considered severe and 74 deaths were confirmed. Although neurological manifestations were frequently reported among severe cases, cardiovascular failure was the most frequent reported cause of death, occurring in 77% of the cases [46]. In a study of 68 fatal cases, autopsy identified respiratory and cardiac failure as the main cause, with myocarditis occurring in 36.6% of the fatal cases [40]. In a systematic review, Cerbino-Neto et al. identified 2140 deaths associated with CHIKV infection. Despite the limited information on clinical manifestations, cardiovascular failure was ranked the third most frequent cause of death, occurring in 42% of cases. This was slightly lower than the rates of neurological dysfunction (47.4%) and respiratory dysfunction (43.9%) [47]. In another systematic review, cardiovascular manifestations were considered the most frequent atypical presentation, accounting for 54.2% of severe cases, with cardiac rhythm disturbances reported in 52% of cases, heart failure occurring in 15%, pericarditis in 5%, and acute myocardial infarction in 2%. The estimated mortality rate was 10% for cardiovascular complications, potentially reaching 20% in patients with other comorbidities [48]. In a recent review, Traverse et al. identified arrhythmia as the most prevalent reported manifestation, but also noted a wide range of other manifestations including congestive heart failure, acute myocardial infarction, myocarditis, and pericarditis. The mortality of hospitalized patients varied between studies from 26% to 48%. The worsening of pre-existing conditions such as hypertension, ischemic coronary disease, and heart failure were reported in some studies as causes of hospitalization [20].

Despite the association of comorbidities worsening, studies show that patients without previous cardiovascular disease can also develop acute cardiac impairment [14,33,49]. In Réunion Island between 2005 and 2006, Lemant et al. evaluated 33 cases of patients admitted to intensive care units (ICU), and only 18% were due to the decompensation of pre-existing disease [49]. Crosby et al. described 65 patients in ICU during the 2013–2014 epidemics in Caribbean islands and, although 83% had a pre-existing disease (predominantly chronic hypertension, diabetes mellitus, chronic heart failure, and chronic renal failure), only 41.5% of the admissions were due to the decompensation of comorbidities, with most severe cases related to the direct effect of CHIKV infection [33].

In the study by Economopoulou et al. of 610 CHIKV cases classified as atypical, cardiovascular disease accounted for the majority of them (37%). When assessed by type of involvement, the absence of prior cardiovascular disease was noted in 45% of the 84 cases of heart failure, 65% of the 44 cases of arrhythmia, and 63% of the 35 cases of myocarditis, reinforcing that cardiovascular involvement is independent of previous pathological conditions [14]. Given the evidence and significance of cardiac involvement in CHIKV, authors have recommended that physicians remain vigilant for clinical signs of cardiovascular involvement associated with CHIKV infection, especially in endemic areas. Additionally, they advise the use of complementary exams such as electrocardiograms, cardiac enzymes, echocardiograms, and MRIs for early identification of cases to improve outcomes [20,48,50].

## 7. Pulmonary Involvement

Despite the description of severe acute respiratory syndrome (SARS) and respiratory failure associated with CHIKV infection, pulmonary involvement has been described in review articles as a rare event [20,50]. On the other hand, studies involving hospitalized patients with severe forms and fatalities have described pulmonary involvement as a frequent event [14,46,51]. Among the 610 cases of extra-articular manifestations on Réunion Island in 2005, despite the predominance of cardiovascular and neurological complications, respiratory manifestations were the third most frequent cause, occurring in 25% of cases, with pneumonia accounting for 17% and respiratory failure for 8% of the cases. Of the 102 pneumonia CHIKV-associated cases, only 20% had respiratory comorbidities, with 17 cases of chronic obstructive pulmonary disease and 3 cases of asthma [14].

From systematic reviews reporting CHIKV fatalities, respiratory dysfunction was the second most mentioned cause and was documented in 6.4% of 2140 analyzed deaths [47]. Dorléans et al., in an analysis of 74 CHIKV-related deaths, reported respiratory failure in 58.1% of cases, either isolated or associated with failure of other organs [46]. Autopsy studies have also identified pulmonary involvement as a frequent finding. In an analysis of 68 deaths due to chikungunya in Northeast Brazil, dyspnea was present in one-third of the cases, and of the 48 cases subjected to autopsy, cardiac and respiratory failure were the most frequent causes of death, accounting for 76.2% of the cases. Congestion and edema were the most frequent findings, with pneumonitis occurring in 52.4% of cases and pulmonary hemorrhage in 57.1% [40].

## 8. Renal Involvement

Renal involvement associated with CHIKV infection has been reported as a significant cause of disease complication, with a varied spectrum usually with injury from pre-renal damage related to shock or immune-mediated injury. In a review of 54 articles reporting renal involvement in CHIKV, Costa et al. reported a frequency of renal injury in hospitalized patients ranging from 21 to 45%, being higher in atypical and severe forms [52]. In the 2005 epidemic on Réunion Island, among the 610 hospitalized patients, 120 (20%) presented with acute pre-renal kidney failure, with only 41 of them (34%) related to the exacerbation of pre-existing kidney disease [14]. In Guadeloupe and Martinique, 1836 hospitalized patients were identified, 674 classified as atypical and another 354 as severe, with renal alterations occurring in 26% of cases, the third most frequent clinical syndrome [46].

## 9. Comorbidities Associated with Severe Disease and Fatal Outcomes

Despite the evidence of severe disease occurring in the absence of comorbidities, the presence and decompensation of comorbidities have been classically associated with disease worsening and fatal outcomes. In an analysis of 65 patients admitted to the intensive care unit during an epidemic in the Caribbean islands, 83% had a history of comorbidities, but the cause of hospital admission due to decompensation of the underlying disease occurred in 41.5% of cases [33]. In the study by Lemant et al., decompensation of comorbidities as a cause of ICU admission was lower, accounting for 18% of admissions [49].

Simião et al. analyzed 254 deaths in an epidemic that occurred in 2016–2017 in Northeast Brazil. Previous comorbidities such as kidney disease (prevalence rate (PR), 13.9; CI, 7.86–24.76; *p* < 0.001), diabetes (PR, 9.75; CI, 7.25–13.10; *p* < 0.001), and hypertension (PR, 7.65; CI, 5.90–9.91; *p* < 0.001) had a significantly higher prevalence of death compared to cases without comorbidities [36]. In a study by Godaert et al., the authors compared 267 elderly patients with 109 young patients and found that, although comorbidities only partially explained the progression to severe forms, elderly patients with typical CHIKV had less comorbidity than those with severe CHIKV presentation. The authors considered that CHIKV infection could induce acute decompensation of one or more comorbidities, and that the symptoms related to this decompensation might be more evident than those related to CHIKV infection [53].

In the Martinique and Guadeloupe outbreak, a comparison of 1821 non-severe cases with 361 severe cases identified a higher frequency of elderly patients in severe cases (69.5%, *p* < 0.001). Among severe cases, 77% had at least one comorbidity compared to only 46% of non-severe cases (*p* < 0.001) [46]. In a review of the association between chikungunya and diabetes involving 11 articles, Barreto et al. identified hypertension as the primary cause and diabetes as the second leading cause contributing to deterioration, symptom intensity, and hospitalization in CHIKV patients [54].

The simultaneous circulation of multiple arboviruses in the Americas has been documented in recent years, resulting in an increase in co-infection cases [5,55]. Consequently, the introduction of CHIKV into areas where other arboviruses were already circulating has been hypothesized as a potential factor contributing to severe cases. The frequency of co-infections varies widely in the literature. For example, during a CHIKV outbreak in St. Martin, a study by Omarjee R et al., 2014 [56] documented a co-infection rate of 2.5% (16/635), whereas a study by Saswat T et al., 2015 [57] reported a rate of 44.8% (43/96) during a dengue epidemic in India. The most commonly reported co-infections cases involve CHIKV and DENV, likely due to the prolonged co-circulation of these two viruses in various outbreaks. In contrast, dual infections involving CHIKV and ZIKV are less common and have primarily been reported since 2016, coinciding with the rapid spread of these viruses—especially in the Americas—following the Brazilian ZIKV epidemic [55,58,59,60].

A systematic review and meta-analysis reported a prevalence of Zika and chikungunya co-infection ranging from 0.1% to 3.5%, with higher frequencies observed in Haiti, Mexico, and Nicaragua [61]. Similarly, a study on the global prevalence of dengue and chikungunya co-infection estimated it at 2.5%, with significantly higher rates in Colombia (37.4%) and Madagascar (18.2%) [62]. Interestingly, most studies do not associate co-infection with increased disease severity. However, severe cases in the context of co-infection have been reported [57,63,64,65,66]. For example, among 28 cases of dengue and chikungunya co-infection, three exhibited severe manifestations, including hemorrhagic symptoms and central nervous system involvement [57].

During the 2015–2016 Zika epidemic in Colombia, 34 co-infection cases were identified from 23,871 samples, including two fatal DENV-CHIKV cases and five CHIKV-ZIKV cases. Of these, two resulted in fetal deaths, while the remaining cases involved neurological syndromes and sepsis. In another study, Ferreira et al., 2020 analyzed sequential Zika and Chikungunya epidemics in 2015–2016 and found that among 148 neurological cases, 50 (33.8%) involved arbovirus co-infection, with 46 being dual ZIKV-CHIKV infections. Notably, Guillain–Barré syndrome cases with dual infections more frequently required ventilatory support and had longer hospital stays compared to mono-infections. However, the study found no evidence that prior dengue infection led to more severe outcomes than cases without a history of dengue [43]. Conversely, studies comparing patients with mono-infections to those with dual infections have not shown an association between co-infection and severe cases [19,60]. Raza et al. analyzed 291 hospitalized dengue cases in Pakistan and identified 54 (18.5%) cases of dual dengue and chikungunya infections. No significant differences in complications or severe clinical outcomes were found between the groups, although retro-orbital pain and restlessness were more frequent among co-infected patients. Longer hospital stays and greater severity were associated with comorbidities (*p* < 0.001), particularly diabetes mellitus (*p* = 0.004) [67].

In Nicaragua, among 263 patients with arbovirus infections, 71 (20.1%) presented with co-infections, including 43 cases of dengue and chikungunya. No statistically significant differences in clinical manifestations were observed between mono-infection and co-infection cases [60]. In India, Taraphdar et al., 2012 compared 131 chikungunya mono-infections, 104 dengue mono-infections, and 68 co-infections. Co-infections exhibited milder symptoms, with less edema, lower arthralgia intensity (compared to chikungunya mono-infections), and reduced abdominal pain frequency (compared to dengue mono-infections). All co-infected patients recovered quickly [68]. A study of 68 chikungunya-related deaths by de Lima et al., 2021, found that most cases (73.5%) involved mono-infections, suggesting that co-infection does not significantly contribute to mortality [40]. Most studies indicate that co-infections do not significantly increase disease severity compared to mono-infections. Although some severe cases and complications have been documented, they appear to be exceptions rather than the norm, often influenced by underlying comorbidities.

## 10. Association Strength

In a case–control study conducted in Northeast Brazil, Oliveira et al. compared 82 cases of laboratory-confirmed CHIKV fatality with 164 controls and identified heart disease (OR 3.8; CI: 1.53–9.26) and chronic kidney disease (OR 12.77; CI: 2.75–59.4) as factors associated with deaths. Glucose alterations were also associated with a worse prognosis (OR: 13.5; CI: 1.3–135.0; *p* = 0.038). The findings suggest that the decompensation of underlying diseases may contribute to severe cases, which could justify hospitalizations and a prolonged period of days to weeks until the fatal outcome, as suggested by other authors [69]. Although deaths associated with epidemic arboviruses typically occur in the early days of the disease, infections associated with chikungunya can occur later. In the study by Simião et al., an analysis of 254 officially confirmed deaths found that the deaths occurred during the acute (49.0%) and post-acute (45.3%) phases of the disease. Another 5.7% were recorded after 90 days from the onset of symptoms (chronic phase), reinforcing the disease pattern that extends beyond the acute phase [36]. The occurrence of deaths weeks after the acute infection could be one of the reasons justifying the underreporting of chikungunya-related deaths by physicians, who may be unaware of this unusual time frame until a fatal outcome and thus do not report it on death certificates as related to the infection [10,12].

To estimate the risk of death in the presence of infection compared to individuals without the disease, Cerqueira-Silva et al. conducted a population-based matched cohort study and a self-controlled case series from 2015 to 2018 in Brazil. In the matched cohort study, 143,787 individuals diagnosed with chikungunya were included, and the risk of death from all natural causes was assessed compared to uninfected controls. The risk ratio (RR) of death in the infected group was 8.4 (95% CI 4.85–20.18) compared to the unexposed group between 1 and 7 days and persisted between 57 and 84 days, with RR of 3.0 (2.46–3.72), and reduced to 1.41 (0.82–1.35) from 85 to 168 days. When secondary outcomes were evaluated, the risk rate (RR) of death within 28 days was 1.8 (0.58–7.03) for cerebrovascular diseases, 3.74 (1.33–16.93) for diabetes cases, and 3.66 (1.25–13.96) for ischemic heart disease. In the self-controlled case series arm of the study, out of 182,140 officially confirmed chikungunya cases, 1933 were deaths. Similar findings were observed with an incidence rate ratio (IRR) for all-cause mortality within seven days of 8.75 (7.18–10.66), and it remained elevated 57–84 days after disease onset com IIR of 1.59 (1.26–2.00). In the last period, from 85 to 168 days, the IRR was 1.09 (0.92–1.29). For secondary outcomes, the incidence rate ratio of death within 28 days was 2.73 (1.50–4.96) for cerebrovascular diseases, 8.43 (5.00–14.21) for diabetes cases, and 2.38 (1.33–4.26) for ischemic heart disease, with no identified risk beyond 85 days. In the evaluation of subgroups by sex and age, the risk of death was increased in elderly (≥60 years) and male sex in both studies designs [15].

The study’s findings support the initial hypotheses that chikungunya is associated with risks of deaths from heart disease, cerebrovascular disease, kidney disease, and diabetes. The results also reinforce the initial hypothesis that deaths occurred later, unlike other arboviruses, such as dengue, where most of the deaths occur in the acute phase of the disease, a few days after the onset of symptoms. The authors suggest the need for new guidelines to raise awareness among professionals about the risk of deaths beyond the acute phase and provide guidance for evaluating and monitoring cerebrovascular, cardiovascular, metabolic, and renal systems.

## 11. Clinical Guidelines: The Importance Beyond Joint Manifestations

Case classifications of chikungunya in clinical guidelines have been primarily based on the symptoms and duration of joint manifestations, guiding even the clinical management, with little to no emphasis on severe clinical forms or systemic organ involvement. In a recent systematic review of the chikungunya clinical management guidelines worldwide, only 65% of them had a definition of severe disease for chikungunya but only 35% included atypical presentations in their definition [17]. This is partly due to the concept that these forms, although described as atypical, are rare, thus not alerting and contributing to the early detection and management of severe cases, responsible for fatal outcomes and sequelae. Highlighting the relevance of severe forms can also help reduce deaths, aligning with the World Health Organization (WHO) goals of reducing vector-borne fatal outcomes by 50% and 75% by 2025 and 2030, respectively, as published on the global vector control response 2017–2030 (available at https://www.who.int/publications/i/item/9789241512978 (accessed on 1 September 2023)). In 2015, a panel of experts proposed a new classification of chikungunya cases, distinguishing four categories: (1) acute, (2) atypical, (3) severe acute, and (4) chronic. Although the classification proposed in 2015 represents progress, an evaluation conducted in elderly people with chikungunya in Martinique showed that 42.7% of the cases were not adequately classified by the proposed system, suggesting the need for adjustments [28]. Moreover, the term “atypical” refers to something that does not correspond to the normal, an unexpected situation, minimizing its relevance.

Based on recent evidence of the relevance and frequency of severe forms and fatal outcomes, the Brazilian Ministry of Health will incorporate a new case classification and guidelines for diagnosing and managing extra-articular manifestations, including severity gradients. The new guideline will use a classification based on an expanded clinical spectrum and severity grades, which include the following:

1. Chikungunya: an infection case without “extra-articular” manifestations.
2. Chikungunya with extra-articular manifestations: a case accompanied by other extra-articular manifestations: neurological, cardiovascular, dermatological, ophthalmological, hepatic, renal, respiratory, or hematological, among others.
3. Severe chikungunya: a case accompanied by failure of at least one organ or system, threatening the patient’s life and requiring hospitalization.

The Pan American Health Organization, sensitive to these arguments, published a note on changing the disease classification based on these discussions, although it has not yet updated its case definitions, clinical classification, and disease phases for dengue, chikungunya, and Zika (available at https://www.paho.org/en/documents/case-definitions-clinical-classification-and-disease-phases-dengue-chikungunya-and-zika (accessed on 1 September 2023)). A classification system encompassing all aspects of CHIKV can contribute to greater awareness among healthcare professionals about the severity and complications associated with the disease, analogous to what has already been done for other epidemic infectious diseases. Additionally, it allows for the enhancement of the epidemiological surveillance system, ensuring that cases of the disease are reported and investigated more accurately and consistently reflects the disease profile, estimating the frequency of clinical forms, the proportion of severe cases, and the lethality associated with CHIKV cases. This enables the analysis of epidemiological indicators from different locations and comparisons with indicators of other diseases, including arboviruses such as dengue and Zika. A structured classification helps guide the standardization of care for cases according to clinical complexity and severity, guiding actions such as defining the treatment environment based on disease complexity, the use of complementary exams, and treatment.

## Figures and Tables

**Table 1:** Characteristics of epidemics with reports of excess deaths related to CHIKV.

Author	Country (Region)	Population (Estimated)	Outbreak Year	Outbreak Peak (Months)	Excess Mortality	Associated Virus Strain	Vector
Beesoon, 2008 [25]	Republic of Mauritius (Indian Ocean)	1,250,000	2006	March to May	(1) The number of deaths increased by 16.7% (95% CI 11.0–23.5%) from January to June 2006 compared to the number of deaths in 2005. (2) Excess deaths: 743 for the months of March to May, coinciding with the peak of the CHIKV epidemic.	Indian ocean lineage (IOL)	*A. albopictus*
Mavalankar, 2008 [24]	India (Ahmedabad)	5,637,000	2006	August to September	(1) Mortality rates in August, September, and October 2006 increased by 22%, 57%, and 33%, respectively, compared to the average mortality rates for these months from 2002 to 2005, coinciding with the peak of the CHIKV epidemic. (2) Excess deaths: 3056 in 2006. (3) At the peak of the epidemic in September: 1448 excess deaths.	Indian ocean lineage (IOL)	*Ae. aegypti*
Josseran, 2006 [26]	Réunion Island	770.00	2005–2006	January to February	(1) Crude death rates (CDRs) from January to April 2006 were 7.1%, 34.4%, 25.2%, and 8.3% higher, respectively, than the expected rates compared to the average rates for these months from 2002 to 2004 (*p* < 0.01 for February and March). (2) Excess deaths: 260. (3) Estimated case fatality rate: ≈1/1000 cases.	Indian ocean lineage (IOL)	*A. albopictus*
Freitas, 2019 [27]	Jamaica	2,720,554	2014	August to November	(1) Excess deaths: 2499 deaths in 2014, above the average of the two years prior to the epidemic. (2) Overall increase in mortality: 91.9 per 100,000 inhabitants. (3) Strong positive correlation between the monthly incidence of chikungunya and excess deaths during the epidemic period (Spearman Rho = 0.939; *p* < 0.005).	Asian lineage	*Ae. aegypti*
Freitas, 2018 [23]	Puerto Rico	3,560,010	2014	August to October	(1) Excess deaths: 1310 concurrently with the peak of the 2014 chikungunya epidemic compared to the two previous years. (2) Estimated chikungunya mortality rate: 30.1 deaths per 100,000 inhabitants.	Asian lineage	*Ae. aegypti*
Freitas, 2018 [21]	Guadalupe and Martinique	Guadalupe 400,186 Martinique 383,911	2014	April to November	(1) Excess deaths: 639, compared to the three previous years. (2) There was a strong correlation between monthly CHIKV incidence rates and excess deaths (R = 0.81, *p* < 0.005). (3) Estimated excess mortality rate: 81.5 deaths per 100,000 inhabitants, and a case fatality rate of 2.08 deaths per 1000 CHIKV cases in 2014.	Asian lineage	*Ae. aegypti*
Freitas, 2018 [22]	Dominican Republic	10,282,115	2014	April to November	(1) Excess deaths: 2853 compared to the years 2011–2012. Adjusting for estimated underreporting, excess deaths totaled 4952 during the 2014 epidemic. (2) Estimated mortality rate: 28.9 deaths per 100,000 inhabitants; adjusting for underreporting: 49.8 deaths per 100,000 inhabitants. (3) Strong correlation between monthly excess deaths and chikungunya cases (Pearson’s r = 0.89).	Asian lineage	*Ae. aegypti*
Brito, 2017 [10]	Brazil/Pernambuco State	~9500.00	2016	January to April	(1) Excess deaths: 4235 deaths in the state of Pernambuco in 2016 compared to the average of the four previous years. (2) During the first four months, coinciding with the peak of the CHIKV epidemic, 2919 excess deaths were recorded, with increases of 33% in January, 48% in February, 66.1% in March, and 40% in April.	East-Central-South African (ECSA)	*Ae. aegypti*
Freitas, 2017 [13]	Brazil (three states)	Pernambuco 9,410,773 Rio Grande do Norte 3,474,998 Bahia 15,498,733	2015–2016	January to April	(1) Excess deaths compared to the years 2011 to 2013 for the four states: Pernambuco (4505); Rio Grande do Norte (1478); Southern Bahia (546); Northern Bahia (339). (2) The excess mortality rates per 100,000 inhabitants were Pernambuco (47.9), corresponding to a 21% increase in deaths; Rio Grande do Norte (42.5); Southern Bahia (32.0); and Northern Bahia (30.0).	East-Central-South African (ECSA)	*Ae. aegypti*
